# Inter- and intrapopulation variation in the response of tree seedlings to drought: physiological adjustments based on geographical origin, water supply and species

**DOI:** 10.1093/aobpla/plx037

**Published:** 2017-07-29

**Authors:** Felipe S Carevic, José Delatorre-Herrera, José Delatorre-Castillo

**Affiliations:** 1 Laboratory of Plant Ecophysiology, Faculty of Renewable Natural Resources, Arturo Prat University, Iquique, Chile

**Keywords:** Atacama Desert, drought stress, *Prosopis*, reforestation, seedling provenances, water relations

## Abstract

Initiatives to restore natural ecosystems have had little success in arid and hyperarid ecosystems. In this context, the natural seedling establishment is particularly affected by drought patterns and climatic variability. Likewise, the effect of plant provenance on forest restoration success remains unclear, although previous studies have concluded that some seed locations might be better able to tolerate water stress. In this study, we examined the physiological mechanisms involved in the drought stress resistance of *Prosopis tamarugo* and *Prosopis alba* seedlings from different arid and hyperarid locations of the Atacama Desert in northern Chile. We measured the xylem water potential (Ψ), cuticular transpiration (E_c_), specific leaf area (SLA) and pressure–volume curves at the intrapopulation and interpopulation levels of seedlings of both species subjected to three drought-induced treatments. In addition, plant characteristics such as seedling height (Sh), stem diameter (Sd), leaf biomass (Lb), root biomass (Rb) and seedling survival (Ss) were measured during the treatments. Seedlings of most hyperarid habitats had the highest values of Ψ and water content relative to the turgor loss point, as well as decreased SLA, especially during the strongest drought treatment. Ψ was strongly correlated with Sh in both species, and soil humidity was correlated with Sd. This study highlights the high variability of physiological responses to water stress in both species at the interpopulation and intrapopulation levels, which provides us with a powerful seed selection tool for future reforestation programmes aimed at the early selection and genetic improvement of species of the *Prosopis* genus.

## Teaser

Actions aimed at reforesting arid areas around the world are increasingly complex. In our study, we evaluated the physiological resistance of Prosopis (mesquite) seedlings from different collection sites in the Atacama Desert, northern Chile. Our results show that seedlings of the most hyperarid habitats had the highest tolerance to water stress, as compared to seed collected from less arid sites. These findings are useful for increasing knowledge of the endogenous ecophysiological behaviour of Prosopis spp., which will be instrumental to elaborate upon well-planned forestation plans directed at regenerating and preserving these endangered woodlands.

## Introduction

In general, drought stress in plants has been identified as a major limiting factor of growth and yield in arid and semiarid ecosystems. Water stress caused by summer drought is the main factor limiting the establishment of this species (Villagra *et al.* 2010). Physiologically, these limitations involve not only stomatal closure but also a decrease in stem water potentials (Ψ) and lower rates of carbon assimilation (Delatorre *et al.* 2008). Moreover, some species prone to water stress during periods of greater water availability tend not to control water loss to the same degree and hence are termed ‘water-spender species’ because they preserve water during dry periods but not during wet ones (Levitt 1980). Conversely, other species tolerate drought stress based on a ‘water conservative strategy’ by avoiding water loss during dry and wet periods adjusting cuticular transpiration (E_c_) and stomatal closure (Villar-Salvador *et al.* 2004). However, several authors agree that the main physiological drought adaptation strategy of arid climate plants seems to be the need to maintain a high water content and cell turgor, which might have a direct effect on their subsequent growth and seed production processes (López *et al.* 2013; Villalobos and Peláez 2015). Nevertheless, not all plants have the same strategies to respond to periods of water stress. For example, greater reductions in the growth of some species facing water limitations, such as *Prosopis alpataco* and *Prosopis argentina*, are affected in a negative way; however, their specific leaf areas (SLAs) are not significantly affected (Villagra and Cavagnaro 2006). On the other hand, under these same water limitations, the SLA of *Erythrina velutina* individuals—a member of the Fabaceae that can tolerate water stress—is significantly affected after long periods of drought stress (Da Silva *et al.* 2010).

*Prosopis* species are widely distributed in natural ecosystems in Africa, America and Asia and have important economic value due to their use as a source of food, seeds and agroforestry (Giordano *et al.* 2011). In the Atacama Desert in northern Chile, individuals of the endangered phreatophyte tamarugo tree (*Prosopis tamarugo*)—endemic to the Tarapacá region—are widely distributed in these arid lands, and, along with white mesquite (*Prosopis alba*) and Chilean mesquite (*Prosopis chilensis*), they are the most common phreatophyte species in the natural ecosystem known as ‘Pampa del Tamarugal’, which is considered an agroforestry system (Ormazábal 1991; Chiappa *et al.* 1997). Unlike *P. tamarugo*, *P. alba* forests are distributed in ecosystems of Argentina, Uruguay, Chile and Peru, and this species is classified as a vulnerable tree in Chile (Benoit 1989).

One of the main obstacles to the success of reforestation programmes in the Pampa del Tamarugal aquifer relates to the water table, which has shown a decline in the past few decades. This trend is associated with both the mining industry and the use of water by the urban population. The water flux into the aquifer is calculated to be between 880 and 1000 L s^−1^, while the water outflow is estimated to be as high as 4000 L s^−1^ (Calderon *et al.* 2015). Another global challenge of the *Prosopis* agroecosystem is its poor performance in natural regeneration compared with other desert species, due mainly to the endozoochory mechanism, since the rainfall is close to 0.6 mm year^−1^ (Zelada 1986). In this context, there is no general consensus that explains the low natural regeneration rates of individuals of the *Prosopis* genus. Previous studies have concluded that the regeneration rates are extremely low mainly due to periods in which the El niño phenomenon occurs, which induces erratic behaviour in the natural germination of seeds (López *et al.* 2006). However, other factors that could affect the regeneration rates relate to the ecological system, such as the pressure of predation from herbivores, the competition with grasses associated with agroforestry ecosystems of *Prosopis* or the water stress that constantly affects the ecosystem (Bush and Van Auken 1991; Weltzin *et al.* 1998; Shackleton *et al.* 2014).

Initiatives to restore natural ecosystems using mesquite (*Prosopis*) species have had little success in northern Chile ecosystems (Altamirano 2006). The effect of plant provenance on forest restoration success remains unclear, although previous studies have concluded that some seed locations—from the natural dispersal centre of origin—might be better able to tolerate water stress (Cony 1995; Cariaga *et al.* 2005). Thus, there is a need to increase the knowledge of the physiological strategy that seedlings from different locations of species from this genus utilize during periods of drought because an intrinsic feature of *Prosopis* is the high genetic variability of its populations, which might produce different responses to the same type of stress (Cony and Trione 1998; Carevic *et al.* 2015). These parameters are especially interesting because they can be used in reforestation plans when selecting seeds. Additionally, under natural conditions, *Prosopis* individuals are often exposed to periods of low temperature and frost during winter. Studies of water relations in *Prosopis* species of the arid zones of Chile are still emerging, and the physiological mechanism of the adaptation of seedlings to drought remains unclear (Altamirano 2006). A better understanding of the strategies used by different *Prosopis* plants for drought tolerance would help to develop comprehensive models of plant water requirements in arid zones and other drought-prone regions. These responses allow the species to survive and even to maintain some growth under adverse conditions, considering that the plant response depends on the nature of the water shortage that induces physiological responses to short-term changes. The goal of this study was to understand the mechanisms involved in drought stress resistance of *P. tamarugo* and *P. alba* seedlings from different locations. We hypothesized that both species will show different adaptive strategies in relation to their drought resistance and provenance. Furthermore, we hypothesized that the provenances of *P. tamarugo* and *P. alba* in the most driest region (Tarapacá) would be the most tolerant to drought because this zone is the area of the natural dispersion of this species. These findings will provide a theoretical basis for the ecological protection and restoration of the Pampa del Tamarugal basin and the surrounding arid region.

## Methods

### Study site

This research was carried out by collecting seeds of *P. tamarugo* and *P. alba* from nine random natural distribution zones of the Atacama Desert (Table 1). The Tarapacá region was the first area from which seeds were collected, with a total of five locations; in the second area, the Antofagasta region, four locations were selected for this purpose. In total, we were able to collect seeds of *P. tamarugo* from eight locations (five in Tarapacá and three in Antofagasta) and of *P. alba* from six locations (two in Tarapacá and four in Antofagasta). Regarding the different collection locations, we were able to identify a hyperarid area of distribution of *Prosopis* forests in which the precipitation does not exceed 0.6 mm year^−1^ (Tarapacá) and an arid zone (Antofagasta) with a precipitation regime that exceeds 35 mm year^−1^.

**Table 1.** Description of each area of collected seeds from the Antofagasta and Tarapaca regions. Data show the collection site, rainfall (mm), spatial geodesic location (WGS 1984), meters above sea level (masl) and seed collected (species).

ANTOFAGASTA REGION					
Site	Mean annual rainfall	UTM E	UTM S	MASL	SC
Quillagua	0.3	443858	7606142	1146	*P. tamarugo/P. alba*
Calama	2.8	505233	7511795	2342	*P. alba*
Chiu-Chiu	5.5	536568	7529509	2533	*P. tamarugo/P. alba*
Toconao	35.7	601286	7435194	2487	*P. tamarugo/P. alba*

TARAPACÁ REGION					
Site	Mean annual rainfall	UTM E	UTM S	MASL	SC
Zapiga	0.6	407442	7816448	1026	*P. tamarugo*
La Huayca	0.6	438480	7741616	1099	*P. tamarugo*
La Tirana	0.6	430328	7751433	1083	*P. tamarugo/P. alba*
Canchones	0.6	442791	7736712	1125	*P. tamarugo/P. alba*
Llamara	0.5	434803	7657083	1167	*P. tamarugo*

Safe collection zones between January and March of 2011 were selected to carry out the collection of seeds. This collection was achieved by installing fences and setting plastic traps under the crowns of the selected trees in the areas where seeds were collected before the seed dropping period.

### Seedling breeding

Plant breeding was carried out in the Estación Experimental Canchones (EEC) of the Universidad Arturo Prat in northern Chile. After removing the cuticle with H_2_SO_4_, we germinated and transplanted the individual plants from both locations to plastic pots that were 15 cm in diameter and 90 cm deep during July 2011. During the entire period of the test, the plants were placed in a greenhouse with a roof of Raschel mesh so that the plants were exposed to the natural abiotic conditions (Table 2). The light level was 70–80 % of the outside light intensity. The soil used in the experiment was a saline-sandy soil obtained from the La Tirana locality, where *P. tamarugo* and *P. alba* are the dominant species.

**Table 2.** Rainfall (mm) and mean of the minimum and maximum temperatures (°C) at the Estación Experimental Canchones site.

Month	2012		
	Rainfall	*T*_min_	*T*_max_
January	0.6	15.3	22.1
February	0.2	9.2	33.9
March	0	15.9	32.2
April	0	8.2	33.1
May	0	17.6	31.4
June	0	15.4	32.3
July	0.4	13.2	33.3
August	0	15.8	33.6
September	0	18.2	34.0
October	0	25.5	33.8
November	0	21.9	33.6
December	0	13.9	31.1

### Water supply treatments

Each seedling was irrigated for a 1-year period (during the first year of growth) with 2.0 L of well water twice weekly based on full soil water capacity (FSWC). We estimated the FSWC based on moisture retention curves and wilting points following Orfánus (2005) and Delatorre (2008) for *Prosopis* species. The water supply treatments consisted of performing an experiment on plants from both regions (Tarapacá and Antofagasta) that were ~1 year old by decreasing the frequency of weekly irrigation of the individual plants. Thus, we used three watering supply periods: once a week until the FSWC was reached (T1) between 18 July and 20 August, 2012; once every 2 weeks until the FSWC was reached (T2) between 21 August and 23 September, 2012; and discontinuation of watering after this date (T3). Measurements for the last treatment (T3) were performed on 30 October 2012. Thus, a total of 240 *P. tamarugo* seedlings (30 for each of the eight locations) and 210 *P. alba* seedlings (35 for each of the six locations) were used for the test. In addition, we used a control group of seedlings for each species and location that was watered twice a week during the entire study period. Two physiological and morphological measurements were performed during each period (T1, T2 and T3).

Two soil humidity sensors (ECH_2_O®) were installed at a depth of 0.3 and 0.6 m for the plants selected for measurement. All of the sensors were positioned randomly in each treatment. The meteorological data from weather stations near the seed collection areas were available and were used to identify climate variables (absolute minimum and maximum temperatures as well as solar radiation) from the origin locations of the seeds.

### Seedling characteristics

The following morphological measurements were carried out in the plants from each provenance during the entire study: seedling height (Sh), stem diameter (Sd), leaf biomass (Lb), root biomass (Rb) and seedling survival (Ss). Sh, Sd and Ss were measured in 28 individuals per treatment on a weekly basis. Seedling height was measured (in mm) with a ruler from the root collar to the terminal bud or to the highest point with green foliage for seedlings that had experienced dieback. Stem diameter (in mm) was measured with callipers at 0.5 cm above the root collar. Seedling survival (%) was quantified during each treatment by noting if the seedling was alive or dead. Resprouted individuals classified as dead were reclassified as alive. Dry biomass of leaves and roots (in g) was measured in 15 individuals per treatment per species in an oven at 65 °C during a period of 48 h. Root and leaf biomass was recorded after oven-drying at 65 °C for 48 h.

### Measures of plant water variables

During the entire study, starting in July 2011, the water potential (Ψ) and cuticular transpiration (E_c_) measurements were carried out. The E_c_ rates provided an estimate of the water that transpired when the stomata were closed, therefore allowing a leaf epidermis permeability assessment (Fernández *et al.* 2015). For these measurements, a homogenous quantity of 11 plants was randomly selected per location/species. Measurements of Ψ were performed using a pressure chamber (Model 1000, PMS Instruments, Corvallis, OR, USA) in mature leaves collected at dawn. The leaves were then placed into plastic bags, taken to the laboratory inside portable coolers, placed in the dark and refrigerated, and then measured immediately.

To measure E_c_, two healthy and fully grown leaves were selected from the same individuals and on the same sampling dates as the water potential measurements. After the leaves were selected, they were taken to the laboratory in a cooler inside glass tubes with the base of the short petiole submerged in water and were left in complete darkness for 24 h at 4 °C to be completely hydrated. Before performing the measurements, the leaves remaining inside the tubes were placed in an illuminated area of the laboratory to facilitate stomatal opening and temperature equilibration (Cape and Percy 1996). Cuticular transpiration was estimated using the free transpiration method (Quisenberry *et al.* 1982; Carevic *et al.* 2010). For this purpose, the leaves were placed on a plastic grille at 5 cm above the laboratory table. Next, their fresh weight was measured on a scale (±0.1 mg) at 5-, 10-, and 30-min intervals for ~7 h. These data were used to create transpiration decline curves for each leaf. The relative water content at the point of stomatal closure (RWC_c_) and cuticular transpiration (E_c_, µmol H_2_O kg^−1^ s^−1^) were deduced from these curves. Then, after being placed in a heater at 65 °C until constant weight was reached, the surface area and dry weight were determined. The SLA (m^2^ kg^−1^) was also calculated for each leaf.

### Pressure–volume curves

To create *pressure*–*volume* curves, a total of two twigs were taken from the same individuals as those used for Ψ and E_c_. These twigs were selected from the seedlings and kept in a refrigerated chamber (3 °C) in darkness. The base of the twig was submerged in distilled water, and the twigs were covered with a polyethylene bag for at least 24 h to facilitate hydration until saturation occurred (Larcher 2003). Data collection was carried out using the free transpiration method (Robichaux 1984), which consists of measuring the Ψ and fresh weight (FW) of the twig over short periods of time and at a constant temperature of 25 °C (±2 °C) until dehydration occurs. Afterwards, the twigs were placed in an oven at 70 °C to obtain their dry weight (DW) and relative water content (RWC = 100[FW − DW]/[FW_sat_ − DW]), where FW_sat_ is the fresh weight after water saturation. Once the *pressure*–*volume* curves were represented in a graph and it was established that there were no oversaturation points, the components of Ψ (osmotic component and turgor or pressure component) could be determined for any hydration status. In addition, the following parameters were determined according to the methodology suggested by Pearcy *et al.* (1989): osmotic potential at full turgor (Ψπ_100_), osmotic potential at zero turgor (Ψπ_0_), water content relative to turgor loss point (RWC_0_) and relative apoplastic water content (RWC_a_).

### Data analysis

We used a completely randomized experimental design with two fixed factors: seed origin and level of water supply (T1, T2 and T3). To evaluate the differences among origin locations and treatment effects regarding the morpho-physiological features measured (SLA and water parameters), repeated-measure ANOVA was used. To test the differences on the effect of water supply treatments on root and leaf biomass between species, an ANOVA test was performed. Normality and equality of variances were appropriately verified through the Kolmogorov–Smirnov test, and the data did not need to be transformed. When differences between subgroups were significant (*P* ≤ 0.05), individual measurements were analysed using Tukey’s HSD test. Likewise, we evaluated possible relationships between physiological parameters by performing regression analyses. All statistical analyses were performed with SPSS software (Version 17.0, SPSS®, Chicago, IL, USA).

## Results

**Figure 1.**
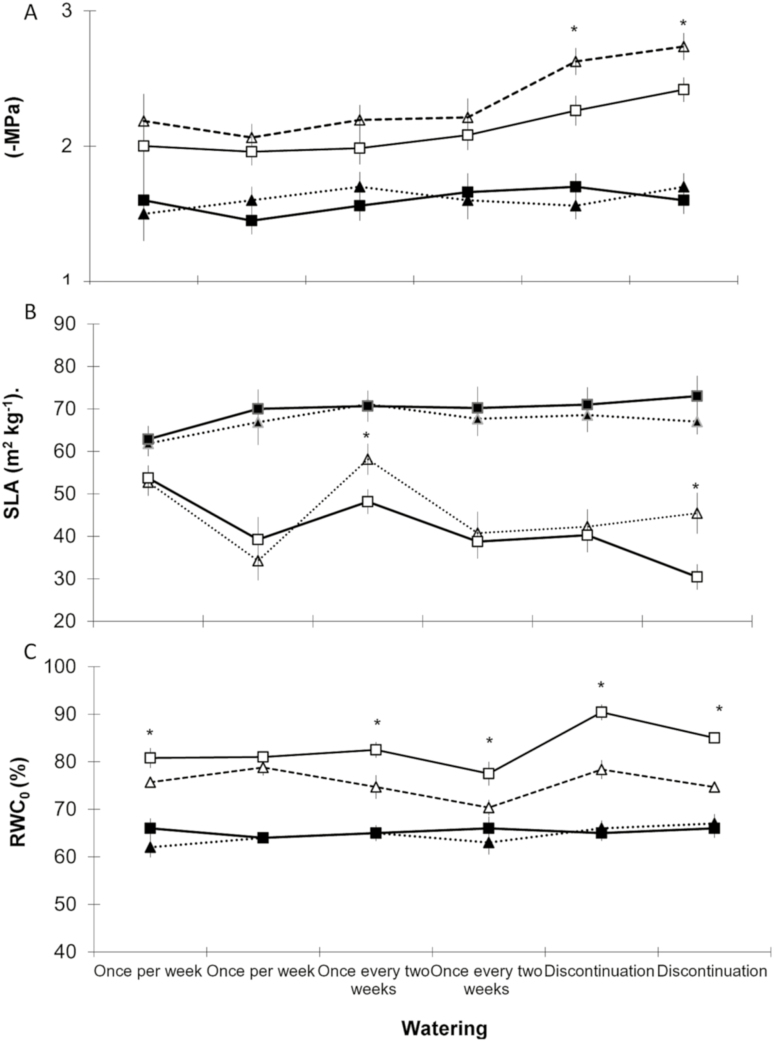
Comparison among provenances of *P. tamarugo* in terms of xylem water potentials (A), SLA (B) and water content relative to turgor loss point (C) parameters. Provenances of Tarapacá and Antofagasta are denoted by white squares and white triangles, respectively. Control seedlings are represented by black figures. Watering supply treatments are denoted in x axis, each with two measurements (see text for information). For each watering supply treatment, statistically significant differences are represented by an asterisk.

Differences between pressure–volume curves during the study period were significant only for RWC_0_ parameter estimated for *P. Tamarugo* at interpopulation level (Fig. 1). Therefore, we mainly focused on analysing the differences found between the other physiological and morphological parameters as well as the possible effect of treatments on water status.

### At interpopulation level for *P. tamarugo*

Figure 1 shows the differences found between locations of *P. tamarugo*. In general, the significantly different parameters between the two locations (the Tarapacá and Antofagasta regions) for *P. tamarugo* included Ψ, SLA and RWC_c_. Regarding the water potentials, significant differences occurred during the last measurement date, when the most aggressive drought treatment had already been applied. In this context, the minimal water potentials reached on that date were 2.41 ± 0.19 MPa for locations from the Tarapacá region and 2.73 ± 0.07 MPa for locations from the Antofagasta region. Regarding SLA, the only differences between the two locations were detected during T2 at the end of the most severe drought treatment (T3). The extreme values ranged from 59.3 ± 0.02 to 31.02 ± 0.02 m^2^ kg^−1^.

The other parameter that registered significant differences was the relative water content at the point of stomatal closure (RWC_0_). The case regarding the values of the relative water content was the most evident. Differences started in T1, T2 and T3 (Fig. 1). Locations from Tarapacá always presented higher values than locations from Antofagasta, whose values never exceeded 80 %. During T1, significant differences were registered for the Sh (*F*_3,108_ = 11.876; *P* < 0.01) and Lb (*F*_3,108_ = 39.801; *P* < 0.001) parameters between populations from La Huayca and Toconao. The mortality rate at the end of the most aggressive drought treatment did not show significant differences, with rates being 68.3 % for Tarapacá and 66.2 % for Antofagasta (*F*_3,108_ = 36.78; *P* = 0.124).

### At intrapopulation level in *P. tamarugo*

The intrapopulation analysis of *P. tamarugo* only showed significant differences in populations from Zapiga and La Tirana in Tarapacá and in Toconao in Antofagasta. The physiological parameters that showed differences were registered only at T3 in both regions (Table 3) and included the SLA, RWC_c_ and Ψ parameters. At the morphological level, during T3, the Ss parameter showed statistically significant differences in the populations of Salar de Llamara and La Tirana (*F*_3,108_ = 231.876; *P* < 0.001; *F*_3,108_ = 189.760; *P* < 0.001, respectively).

**Table 3.** Intrapopulation parameters of *P. tamarugo*. Statistically significant differences (*P* < 0.05) were registered when T3 was applied.

Parameter	La Huayca		Zapiga		Toconao	
	*F*_3,95_	*P*	*F*_3,95_	*P*	*F*_3,95_	*P*
SLA (m^2^ kg^−1^)	9.046	0.006	3.310	0.014	3.099	0.010
RWC_c_ (%)	19.555	<0.001	1.035	0.411	2.222	0.0435
Ψ (MPa)	0.444	0.511	14.680	<0.001	10.098	<0.001

### At interpopulation level for *P. alba*

**Figure 2.**
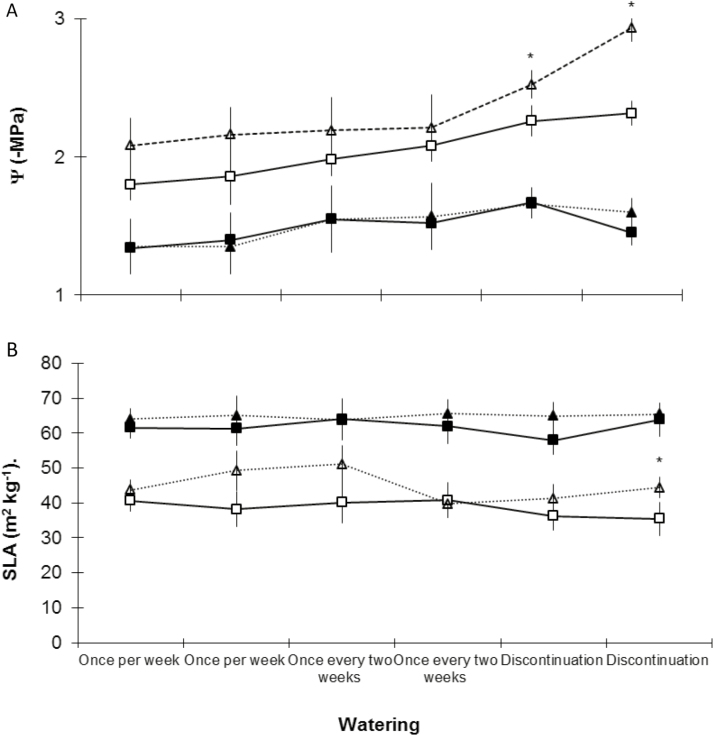
Comparison among provenances of *P. alba* in terms of xylem water potentials (A) and SLA (B). Provenances of Tarapacá and Antofagasta are denoted by white squares and white triangles, respectively. Control seedlings are represented by black figures. Watering supply treatments are denoted in x axis, each with two measurements (see text for information). For each watering supply treatment, statistically significant differences are represented by an asterisk.

In general, the significantly different parameters between the two locations (Tarapacá and Antofagasta regions) for *P. alba* were the xylem water potential and SLA. The xylem water potential showed significant differences when the T3 treatment was applied (Fig. 2). Extreme values from the two locations ranged from −1.8 ± 0.11 to −2.9 ± 0.03. Regarding the SLA parameters, the trends showed higher values in locations from the Antofagasta region than locations from the Tarapacá region. At the morphological level, Sh showed differences between the Toconao and La Tirana locations during T1 (*F*_3,108_ = 122.765; *P* < 0.023). The mortality rate at the end of the most aggressive drought treatment did not show significant differences, with values of 76.3 % for Tarapacá and 58.2 % for Antofagasta (*F*_3,108_ = 87.78; *P* = 0.012).

### At intrapopulation level in *P. alba*

At the intrapopulation level, there were noteworthy differences in the estimated physiological parameters relative to E_c_, SLA, RWC_c_ and Ψ. Statistically significant differences were registered only during T3 in two populations from Antofagasta and one from Tarapacá (Table 4). Regarding the morphological parameters, seedling height was the only one that showed a significant difference during T3.

**Table 4.** Intrapopulation parameters of *P. alba*. Statistically significant differences (*P* < 0.05) were registered when T3 was applied.

Parameter	Quillagua		Chiu-Chiu		La Huayca	
	*F*3,95	*P*	*F*3,95	*P*	*F*3,95	*P*
E_c_ (µmol H_2_O kg^−1^ s^−1^)	0.03	1.345	11.086	<0.001	10.345	<0.001
SLA (m^2^ kg^−1^)	9.111	<0.001	2.123	0.023	1.000	0.657
RWC_c_ (%)	16.432	<0.001	11.111	<0.001	9.456	<0.001
Ψ (MPa)	0.564	0.531	10.598	<0.001	0.009	0.987
Sh (mm)	22.765	<0.01	3.456	0.963	19.875	<0.01

### Relationships among parameters

**Figure 3.**
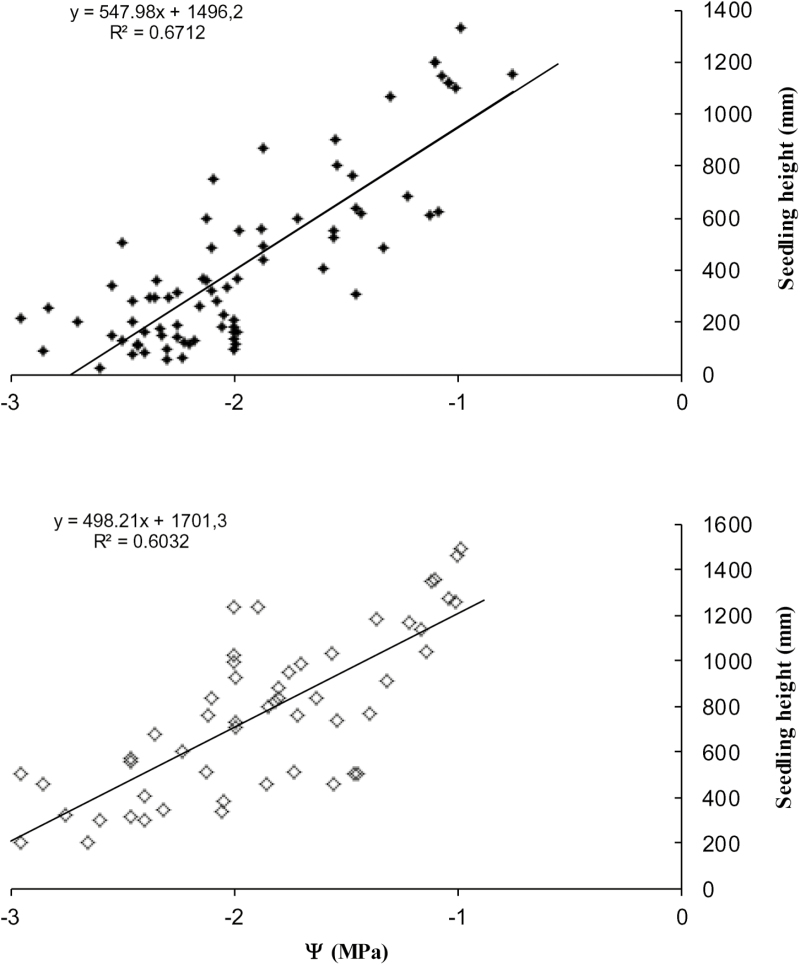
Relationship between xylem water potentials (Ψ) and seedling height for *P. tamarugo* (black figures) and *P. alba* (white figures).

**Figure 4.**
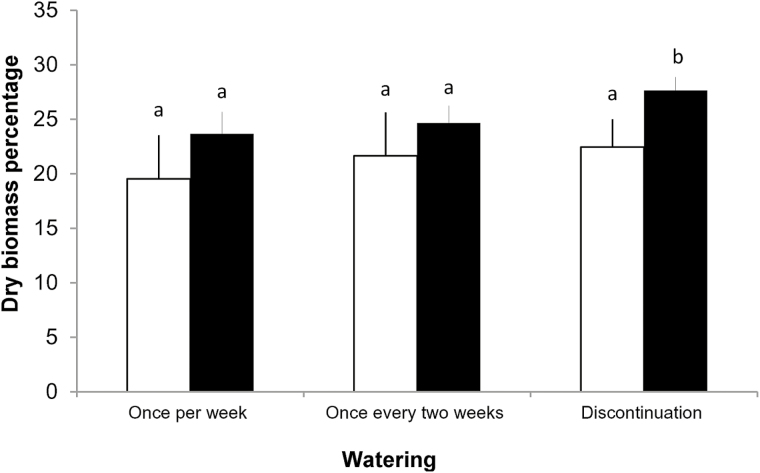
Dry biomass of leaves of *P. tamarugo* (white columns) and *P. alba* (black columns) during each watering treatment induced. Differences are denoted by different letters.

The parameters that showed relationships in both species (*P* < 0.01) were the height of individuals and Ψ measured during T1 (Fig. 3). Likewise, both species registered a positive relationship between soil humidity and plant stem diameter during T1 (*R*^2^ = 0.57, *F*_3,108_ = 124.54; *P* < 0.01; *P. tamarugo* and *R*^2^ = 0.59, *F*_3,108_ = 214.65; *P* < 0.001; *P. alba*) and between Ψ and E_c_ (*R*^2^ = 0.74, *F*_3,95_ = 309.24; *P* < 0.001; *P. tamarugo* and *R*^2^ = 0.79, *F*_3,95_ = 314.01; *P* < 0.001; *P. alba*). The soil humidity ranged during the experiment (physiological measures) between 43 and 45 %. No statistically significant differences were found between the dry biomass of roots in drought treatments and control groups in both species. However, statistically significant differences were found between the dry biomass of leaves in treatments between both species (Fig. 4).

## Discussion

In the present study, we found strong evidence of higher xeromorphism in tamarugo (*P. tamarugo*) than in white mesquite (*P. alba*) at the species level, denoted by a lower ‘Ψ’ and the lowest values of dry leaf biomass obtained during drought treatment experiments. These physiological characteristics could explain the prevalence of *P*. *alba* in more mesic habitats found in the arid to semiarid ecosystems of South America. These results are in accordance with those of other studies which reported stronger effects of decreased water supply on the morphological and physiological parameters of mesic *P*. *alba* than those of other xeric species (Vilela *et al.* 1996; Vilela *et al.* 2003). In terms of provenance, the clear differences obtained for parameters Ψ and SLA showed that the seeds which had originated north of the collection areas included in the present study were well adapted to drought. Thus, the tendency of *P*. *tamarugo* (originating in Tarapacá) to maintain high Ψ and RWC_0_ values during T3 (−2.4 MPa and 90 %, respectively) clearly indicates the presence of an adjustment strategy to withstand stress periods. These results from Tarapacá may be associated with the fact that this distribution area is the natural dispersal centre for this species (Calderon *et al.* 2015), which may have conferred critical ontogenic features to this species in order to adapt to the stress encountered in the Pampa del Tamarugal plain. Other studies which focussed on drought-tolerant species support the idea that seeds originating in natural dispersal areas for the species have genetic and morphological features that positively affect adaptation to the most severe environmental stresses typical of their place of origin (Cony 1996; Sánchez-Vila and Retuerto 2007; Andivia *et al.* 2012).

SLA is a parameter that varies depending on the water stress conditions that affect leaf growth, location of the crown (in terms of light) or nutrient availability (Mousseau 1999; Larcher 2003). In the present study, the origin location values of *P*. *tamarugo* and *P*. *alba* showed significant differences; these differences mainly appeared during the most severe drought treatments, with evidently higher values in the most humid locations (Antofagasta). This strategy may have been linked to a higher accumulation of non-structural leaf biomass in the driest locations (Tarapacá) as a response to reduce excessive water loss through the epidermis, which would allow the plants at this location to maintain higher RWC_0_ values (Larcher 2003). This is a typical strategy of drought-tolerant species and can be used as an effective indicator of the degree of tolerance among species or their offspring. Similarly, in the same study area, Carevic *et al.* (2015) found that increase in leaf biomass is associated with SLA in mesquite (*Prosopis burkartii*) during stress periods caused by frost, which indicates the sensitivity of this parameter to the presence of environmental stress.

Conversely, RWC_0_ has been identified as an important indicator of the water status of leaves under drought conditions (Koide *et al.* 1989). RWC_0_ is strictly related to cell volume, and thus, it reflects the balance among the water content, water supply to the leaf and transpiration rate more accurately (Andivia *et al.* 2012). In the present study, the trends clearly indicated a water conservation strategy during the highest drought period, with maximum values close to 90 % in *P*. *tamarugo*. This strategy better outlines the way in which this species resists drought, which involves retaining a high percentage of water within its leaf tissues before stomatal closure. In *P*. *alba*, drought strategies were mostly reflected by higher accumulation of biomass at the moment of T3 application (indicated by decreased SLA values) and by maintenance of higher water potentials compared with others in more humid locations (Toconao and Chiu Chiu). In contrast, in the semiarid ecosystems of Argentina and under saline stress, *P*. *alba* utilizes strategies that are mainly associated with the suppression of its photosynthetic apparatus rather than those associated with water variables. This is because when faced with such a stress, *P*. *alba* possesses the ability to decrease its net photosynthesis and stomatal conductance at the expense of its Ψ, which decreases as stress becomes more severe (Meloni 2014). In contrast, when we compared Ψ of both the species during the most severe drought treatment, we observed that the extreme minimum values obtained in *P*. *alba* reached values close to −3.0 MPa, whereas *P*. *tamarugo* maintained extreme values of −2.4 MPa during T3. This fact is directly linked to the most efficient stomatal closure and lower E_c_ rate under minimal Ψ values, which was indicated by the positive relationship between Ψ and E_c_ in both species.

It is important to note that the relationships observed between plant height of both species and Ψ afford this parameter a significant influence that inhibits growth during the first months of seedling growth. Thus, maintaining values close to −1.0 MPa benefitted growth even more when seedlings of both species with values close to −3.0 MPa had a lower height. Similar trends were described by Vilela *et al.* (1996) in *Prosopis* spp. with regard to different watering rates, which directly affected the height and stem growth diameters of seedlings. The high variability of physiological response to water stress reported in the present study provides us with a powerful seed selection tool for species that are currently classified as endangered (*P*. *tamarugo*) and vulnerable (*P*. *alba*) by Chilean environmental agencies (Carevic *et al.* 2015). All of these responses, detectable at an age as early as 1 or 2 years, have an advantage that allows their use in reforestation and preservation programmes and represent an important source of information for future programmes aimed at early selection and genetic improvement of *Prosopis* spp.

## Conclusions

In this research, we analysed the effects of different drought treatments on seedlings of two phreatophyte *Prosopis* spp. which grow in hyperarid climate. In general, Ψ, SLA and RWC_0_ underwent rapid change under drought stress. These results indicate that in *Prosopis* spp. individuals, these physiological variables can be used as sensitive indicators for measuring drought stress and are good tools for investigating differences in physiological behaviour under different water supply periods. In addition, these results are useful for increasing the knowledge of the endogenous ecophysiological behaviour of *Prosopis* spp., which will be instrumental to elaborate upon well-planned forestation plans directed to regenerate and preserve these endangered woodlands. Nevertheless, further studies are required to elucidate the genetic mechanism of drought tolerance differences at interpopulation and intrapopulation levels in *Prosopis* spp. Thus, this intriguing and controversial topic warrants focus in future studies.

## Sources of Funding

The authors acknowledge the financial support of FONDECYT project number 11130242 and CONAF project number 047/2011.

## Contributions by the Authors

F.S.C. conceived the idea, performed the physiological measures and wrote the article; J.D.H. performed the statistical analyses; J.D.C. analysed the data and revised the article.

## Conflict of Interest

None declared.
